# Monitoring tafamidis treatment with quantitative SPECT/CT in transthyretin amyloid cardiomyopathy

**DOI:** 10.1093/ehjci/jead030

**Published:** 2023-03-07

**Authors:** René Rettl, Tim Wollenweber, Franz Duca, Christina Binder, Bernhard Cherouny, Theresa-Marie Dachs, Luciana Camuz Ligios, Lore Schrutka, Daniel Dalos, Dietrich Beitzke, Christian Loewe, Roza Badr Eslam, Johannes Kastner, Marcus Hacker, Diana Bonderman

**Affiliations:** Division of Cardiology, Department of Internal Medicine II, Medical University of Vienna, Waehringer Guertel 18-20, Vienna 1090, Austria; Division of Nuclear Medicine, Department of Biomedical Imaging and Image-guided Therapy, Medical University of Vienna, Waehringer Guertel 18-20, 1090 Vienna, Austria; Division of Cardiology, Department of Internal Medicine II, Medical University of Vienna, Waehringer Guertel 18-20, Vienna 1090, Austria; Division of Cardiology, Department of Internal Medicine II, Medical University of Vienna, Waehringer Guertel 18-20, Vienna 1090, Austria; Division of Cardiology, Department of Internal Medicine II, Medical University of Vienna, Waehringer Guertel 18-20, Vienna 1090, Austria; Division of Cardiology, Department of Internal Medicine II, Medical University of Vienna, Waehringer Guertel 18-20, Vienna 1090, Austria; Division of Cardiology, Department of Internal Medicine II, Medical University of Vienna, Waehringer Guertel 18-20, Vienna 1090, Austria; Division of Cardiology, Department of Internal Medicine II, Medical University of Vienna, Waehringer Guertel 18-20, Vienna 1090, Austria; Division of Cardiology, Department of Internal Medicine II, Medical University of Vienna, Waehringer Guertel 18-20, Vienna 1090, Austria; Division of Cardiovascular and Interventional Radiology, Department of Biomedical Imaging and Image-guided Therapy, Medical University of Vienna, Waehringer Guertel 18-20, 1090 Vienna, Austria; Division of Cardiovascular and Interventional Radiology, Department of Biomedical Imaging and Image-guided Therapy, Medical University of Vienna, Waehringer Guertel 18-20, 1090 Vienna, Austria; Division of Cardiology, Department of Internal Medicine II, Medical University of Vienna, Waehringer Guertel 18-20, Vienna 1090, Austria; Division of Cardiology, Department of Internal Medicine II, Medical University of Vienna, Waehringer Guertel 18-20, Vienna 1090, Austria; Division of Nuclear Medicine, Department of Biomedical Imaging and Image-guided Therapy, Medical University of Vienna, Waehringer Guertel 18-20, 1090 Vienna, Austria; Division of Cardiology, Department of Internal Medicine II, Medical University of Vienna, Waehringer Guertel 18-20, Vienna 1090, Austria; Division of Cardiology, Department of Internal Medicine V, Favoriten Clinic, Kundratstraße 3, 1100 Vienna, Austria

**Keywords:** SPECT/CT, SUV quantification, tafamidis, transthyretin cardiac amyloidosis, treatment monitoring

## Abstract

**Aims:**

Tafamidis treatment positively affects left ventricular (LV) structure and function and improves outcomes in patients with transthyretin amyloid cardiomyopathy (ATTR-CM). We aimed to investigate the relationship between treatment response and cardiac amyloid burden identified by serial quantitative ^99m^Tc-DPD SPECT/CT. We furthermore aimed to identify nuclear imaging biomarkers that could be used to quantify and monitor response to tafamidis therapy.

**Methods and results:**

Forty wild-type ATTR-CM patients who underwent ^99m^Tc-DPD scintigraphy and SPECT/CT imaging at baseline and after treatment with tafamidis 61 mg once daily [median, 9.0 months (interquartile range 7.0–10.0)] were divided into two cohorts based on the median (−32.3%) of the longitudinal percent change in standardized uptake value (SUV) retention index. ATTR-CM patients with a reduction greater than or equal to the median (*n* = 20) had a significant decrease in SUV retention index (*P* < 0.001) at follow-up, which translated into significant benefits in serum *N*-terminal prohormone of brain natriuretic peptide levels (*P* = 0.006), left atrial volume index (*P* = 0.038), as well as LV [LV global longitudinal strain: *P* = 0.028, LV ejection fraction (EF): *P* = 0.027, LV cardiac index (CI): *P* = 0.034] and right ventricular (RV) [RVEF: *P* = 0.025, RVCI: *P* = 0.048] functions compared with patients with a decrease less than the median (*n* = 20).

**Conclusion:**

Treatment with tafamidis in ATTR-CM patients results in a significant reduction in SUV retention index, associated with significant benefits for LV and RV function and cardiac biomarkers. Serial quantitative ^99m^Tc-DPD SPECT/CT imaging with SUV may be a valid tool to quantify and monitor response to tafamidis treatment in affected patients.

**Translational perspective:**

^99m^Tc-DPD SPECT/CT imaging with determination of SUV retention index as part of a routine annual examination can provide evidence of treatment response in ATTR-CM patients receiving disease-modifying therapy. Further long-term studies with ^99m^Tc-DPD SPECT/CT imaging may help to evaluate the relationship between tafamidis-induced reduction in SUV retention index and outcome in patients with ATTR-CM and will demonstrate whether highly disease-specific ^99m^Tc-DPD SPECT/CT imaging is more sensitive than routine diagnostic monitoring.

## Graphical Abstract

**Graphical Abstract:**
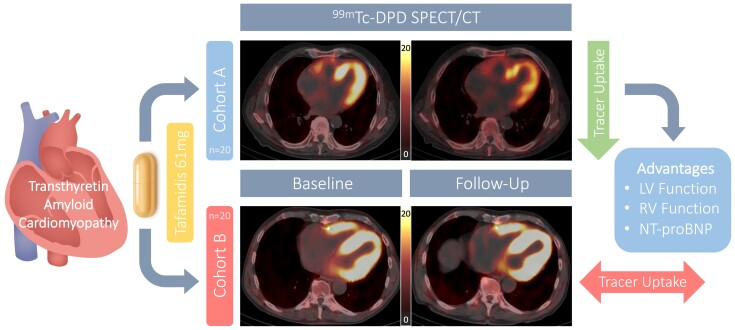
Response to tafamidis treatment and effects on cardiac function and biomarkers. Serial quantitative ^99m^Tc-3,3-diphosphono-1,2-propanodicarboxylic acid (DPD) single-photon emission computed tomography/computed tomography (SPECT/CT) imaging with standardized uptake value (SUV) of representative patients with wild-type transthyretin amyloid cardiomyopathy treated with tafamidis 61 mg once daily. Patients were divided into two cohorts based on the median (−32.3%) of the longitudinal percent change in SUV retention index. *Cohort A* patients had a longitudinal percent reduction in SUV retention index greater than or equal to the median (upper panels), which was associated with significant benefits for left ventricular (LV) and right ventricular (RV) function and serum levels of *N*-terminal prohormone of brain natriuretic peptide (NT-proBNP). *Cohort B* patients had either a percent decrease less than the median or even a percent increase from baseline (lower panels), without significant benefits for LV and RV function and cardiac biomarkers.

**See the editorial comment for this article ‘Expanding the role of bone-avid tracer cardiac single-photon emission computed tomography/computed tomography: assessment of treatment response in transthyretin amyloid cardiomyopathy’, by Sharmila Dorbala, https://doi.org/10.1093/ehjci/jead108.**

## Introduction

The potentially life-threatening diagnosis of transthyretin amyloid cardiomyopathy (ATTR-CM) is pathophysiologically characterized by disintegrated liver-derived transthyretin (TTR) that accumulates as amyloid fibrils in the myocardium, leading to progressive heart failure (HF) with fatal prognosis, especially if left untreated.^1,2^ Once considered a rare disease, novel diagnostic algorithms have led to an increasing number of patients being diagnosed with ATTR-CM in recent years. In particular, nuclear imaging, such as bone scintigraphy with radiolabelled phosphonates like ^99m^Tc-3,3-diphosphono-1,2-propanodicarboxylic acid (DPD), allows visualization of amyloid deposits by planar imaging and has become the non-invasive gold standard for the diagnosis of ATTR-CM.^3^ Novel nuclear imaging techniques, such as single-photon emission computed tomography/computed tomography (SPECT/CT), provide a 3D visualization of radioactivity in the body and determination of a standardized uptake value (SUV) indicating the concentration of the radiopharmaceutical in each tissue^4,5^ and may render additional support in understanding the relationship between treatment and response to disease-modifying therapies like tafamidis,^6^ which positively affects left ventricular (LV) structure and function and improves outcomes in ATTR-CM patients.^7,8^

Hence, the present study aimed to investigate the relationship between tafamidis treatment and response in ATTR-CM patients using quantitative ^99m^Tc-DPD SPECT/CT imaging with SUV to identify nuclear imaging biomarkers that could be used to quantify and monitor response to tafamidis treatment. Therefore, serial ^99m^Tc-DPD scintigraphy and SPECT/CT imaging were performed in ATTR-CM patients prior to and after treatment with tafamidis, divided into two cohorts based on longitudinal changes in SUV parameters, and compared for clinical and imaging outcomes.

## Methods

### Setting

The study was conducted as part of a prospective HF registry at the Department of Internal Medicine II, Division of Cardiology at the Medical University of Vienna, Austria, which includes a dedicated amyloidosis outpatient clinic. The study was approved by the local ethics committee (#796/2010) in accordance with the Declaration of Helsinki, and all participants provided written informed consent prior to registry inclusion for baseline and follow-up assessments.

### Study design

Consecutive registry patients diagnosed with ATTR-CM between June 2019 and April 2021 were screened for study eligibility; eligible study participants underwent baseline and follow-up assessments at our dedicated amyloidosis outpatient clinic as part of a prospective investigative imaging study. ATTR-CM patients were excluded if the following criteria were met: (i) inability to undergo ^99m^Tc-DPD scintigraphy and SPECT/CT imaging at baseline and follow-up; (ii) presence of mutation in the TTR gene (ATTR variant); this patient population is very heterogeneous and has an individual, mutation-dependent and highly variable disease course compared with wild-type ATTR; and (iii) treatment with tafamidis prior to baseline assessment.

### Diagnosis of ATTR-CM

In accordance with the non-invasive diagnostic algorithm published in 2016 by Gillmore *et al.*,^3^ bone scintigraphy was performed in patients with clinical suspicion of cardiac amyloidosis. The diagnosis of ATTR-CM was confirmed when patients had significant myocardial tracer uptake (Perugini grade ≥ 2) on bone scintigraphy and no paraprotein or monoclonal protein was detected by serum and urine immunofixation and serum free light chain assay.^3,9^ Patients with ATTR-CM were offered sequencing of the TTR gene, which was accepted by all patients.

### Tafamidis treatment

Tafamidis 61 mg (a single capsule is bioequivalent to tafamidis meglumine 80 mg^10^) was administered once daily (QD), either under Pfizer’s early access program following publication of the ATTR-ACT trial^8^ or after approval by health insurance reimbursement.

### Clinical and laboratory assessment

Clinical status was defined by New York Heart Association (NYHA) functional class. Submaximal functional capacity was assessed by the 6 min walk test according to the American Thoracic Society guidelines.^11^ Laboratory testing included determination of cardiac biomarkers [*N*-terminal prohormone of brain natriuretic peptide (NT-proBNP)] and troponin T, as well as haemoglobin and serum creatinine levels.

### ^99m^Tc-DPD scintigraphy and SPECT/CT

Nuclear imaging was performed at the Department of Biomedical Imaging and Image-guided Therapy, Division of Nuclear Medicine at the Medical University of Vienna. Planar whole-body images were obtained 2.5 h and SPECT/CT imaging of the thorax 3.0 h after intravenous injection of ^99m^Tc radiolabelled DPD (mean activity: 725.4 MBq ±25.7) using a hybrid SPECT/CT system (Symbia Intevo, Siemens Medical Solutions AG, Erlangen, Germany) equipped with a low-energy, high-resolution collimator [mean dose length product (DLP): 86.3 mGy*cm ±30.7]. There were no differences between baseline and follow-up in the timing of ^99m^Tc-DPD scintigraphy and SPECT/CT imaging after tracer application, ^99m^Tc-DPD activity (725.4 MBq ± 25.7 vs. 720.8 MBq ± 28.7, *P* = 0.452), and DLP (86.3 mGy*cm ± 30.7 vs. 91.9 mGy*cm ± 40.1, *P* = 0.407). Images were acquired in 180° configuration, 64 views, 20 s per view, 256 × 256 matrix, and an energy window of 15% around the ^99m^Tc photopeak of 141 keV. Subsequent to the SPECT acquisition, a low-dose CT scan was acquired for attenuation correction (130 kV, 35 mAs, 256 × 256 matrix, step-and-shoot acquisition with body contour). Image acquisition and reconstruction were performed using xSPECT/CT QUANT (xQUANT) technology (eight iterations, four subsets, 3.0 mm smoothing filter, and a 20 mm Gaussian filter), which uses a 3% National Institute of Standards and Technology (NIST) traceable precision source for standardization of uptake values across different cameras, dose calibrators, and facilities.^12,13^

### Volume of interest

For the automatic contouring of the 3D volume of interest (VOI) of the myocardium, a threshold for maximal activity was developed in phantom experiments at which 39% of maximal activity resulted in a VOI of ∼155 mL, which was in good agreement with the cardiac insert volume of 155 mL and yielded a clear linear relationship between the applied and measured activity concentrations, as confirmed by linear regression analysis (*r* = 0.9998, *P* = 0.010).^6^

### Standardized uptake value

**Figure 1:**
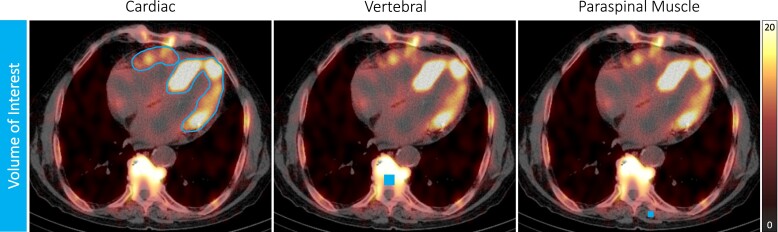
SPECT/CT and volume of interest. SPECT/CT ^99m^Tc-DPD acquisition of a representative treatment-naïve patient with wild-type ATTR-CM showing the VOI for each tissue (VOI cardiac: automatically generated using a threshold-based method, VOI vertebral: 2.92 mL, VOI paraspinal muscle: 1.19 mL) placed to quantify the SUV.

Myocardial uptake on SPECT/CT images was determined using dedicated software (Hermes Hybrid 3D software, Hermes Medical Solutions, Stockholm, Sweden). Three-dimensional myocardial VOIs were automatically generated using a threshold-based method, as previously described, allowing clear separation of myocardial uptake from blood pool activity (*Figure 1*). The myocardial VOIs were reviewed by the operator and corrected if sternal or rib uptake was evident. From these VOIs, an SUV was determined, indicating the concentration of the radiopharmaceutical in the respective tissue, with SUV peak representing the highest average SUV within a 1 cm^3^ volume. Bone uptake (SUV peak vertebral) was calculated by placing a cubic 2.92 mL VOI in an intact vertebral body of a thoracic spine (identical at baseline and follow-up) in an area without degenerative changes to minimize distortion from high degenerative tracer accumulation. For determination of SUV peak paraspinal muscle, a cubic 1.19 mL VOI was placed in the left paraspinal muscle, attempting to match the same position at baseline and follow-up. Confounders caused by competing tracer uptake between respective tissues can be overcome by a composite SUV retention index that balances uptake between the heart, bone, and soft tissue compartments and thus may provide a means of monitoring response to therapy.^14^ It was calculated according to the following formula:


$$\mathrm{SUV}\;\mathrm{retention}\;\mathrm{index}=\left(\frac{\mathrm{SUV}\;\mathrm{peak}\;\mathrm{cardiac}}{\mathrm{SUV}\;\mathrm{peak}\;\mathrm{vertebral}}\right)\times \mathrm{SUV}\;\mathrm{peak}\;\mathrm{paraspinal}\;\mathrm{muscle}$$

### Nuclear imaging analysis

Planar whole-body images were evaluated independently by two experienced physicians and visually graded according to Perugini *et al.*^15^ as previously described. Discrepancies in grading were resolved by consensus. SPECT/CT acquisitions of the thorax were analysed with dedicated software (Hermes Hybrid 3D software, Hermes Medical Solutions, Stockholm, Sweden) and evaluated by two independent, experienced observers blinded to patients’ baseline values. Dedicated software and SUV calculation methods were not changed between baseline and follow-up.

### Transthoracic echocardiography

Transthoracic echocardiography (TTE) was performed by certified and experienced operators using modern equipment (GE Vivid E95, Vivid E9, and Vivid 7, GE Healthcare, Wauwatosa, WI, USA) according to current recommendations.^16,17^ Image analyses were performed after image acquisition on a modern offline clinical workstation equipped with dedicated software (EchoPAC, GE Healthcare, Wauwatosa, WI, USA) by certified cardiologists who were blinded to patients’ baseline values. Dedicated software and calculation methods were not changed during measurements over time.

### CMR imaging

Patients without contraindications [e.g. chronic kidney disease (CKD) stage 4 or 5 with an estimated glomerular filtration rate <30 mL/min/1.73 m^2^, or implanted cardiac device] underwent cardiac magnetic resonance (CMR) imaging on a 1.5 T scanner (MAGNETOM Avanto Fit, Siemens Healthcare GmbH, Erlangen, Germany) according to standard protocols,^18,19,20^ which included late gadolinium enhancement (0.1 mmoL/kg gadobutrol, Gadovist, Bayer Vital GmbH, Leverkusen, Germany) and T1 mapping. All CMR imaging parameters were analysed with dedicated software (cmr42, Circle Cardiovascular Imaging Inc., Calgary, Alberta, Canada) by certified radiologists and cardiologists who were blinded to patients’ baseline values. Dedicated software, T1 mapping, and extracellular volume (ECV) calculation methods were not changed between baseline and follow-up.

### Statistical analysis

Continuous variables are expressed either as mean and standard deviation (SD) or as median and interquartile range (IQR). Categorical variables are presented as numbers and percentages. Analysis of variance (ANOVA), Kruskal–Wallis test, and *χ*^2^ test were used to compare between multiple cohorts, while the paired *t*-test, Mann–Whitney *U* test, and *χ*^2^ test were used to compare between two cohorts. We considered a two-sided significance level alpha = 0.05 for statistical testing. *P*-values are to be interpreted in a descriptive way throughout. All statistical analyses were performed with SPSS version 26 (IBM Corp., New York, NY, USA).

## Results

### Study participants

**Figure 2:**
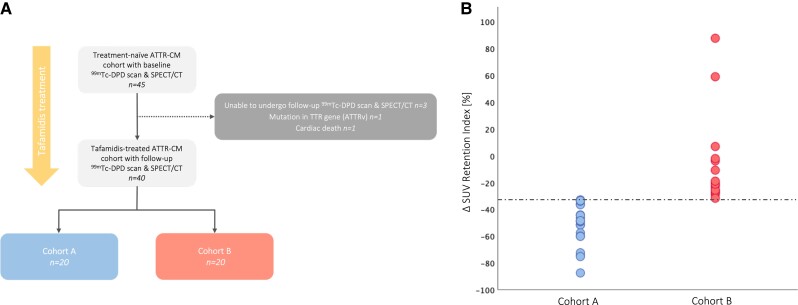
(*A*) Patient flowchart. A total of 45 treatment-naïve patients with transthyretin amyloid cardiomyopathy (ATTR-CM) who underwent ^99m^Tc-DPD scintigraphy and single-photon emission computed tomography/computed tomography (SPECT/CT) imaging were screened for the study. Reasons for study exclusion or discontinuation are depicted. (*B*) Classification based on percentage change in SUV retention index from baseline. Tafamidis-treated ATTR-CM patients were divided into two cohorts (*Cohort A* and *Cohort B*) based on the median percent change in SUV retention index from baseline (−32.3%, dashed line).

A total of 45 treatment-naïve ATTR-CM patients were evaluated for study eligibility, of whom 40 wild-type ATTR-CM patients who underwent ^99m^Tc-DPD scintigraphy and SPECT/CT imaging prior to and after treatment with tafamidis 61 mg QD [median, 9.0 months (IQR: 7.0–10.0)] were deemed eligible; detailed reasons for study exclusion or discontinuation are shown in *Figure 2A.* All study participants (*n* = 40) underwent TTE at baseline and follow-up [median, 8.0 months (IQR: 7.0–11.0)]; in addition, 25 ATTR-CM patients without contraindications were subjected to baseline and follow-up CMR imaging [median, 9.0 months (IQR: 7.0–10.0)]. ATTR-CM patients were divided into two cohorts based on the median (−32.3%) of the longitudinal percent change in SUV retention index. Twenty ATTR-CM patients treated with tafamidis had a longitudinal percent decrease in SUV retention index greater than or equal to the median and were assigned to *Cohort A*, whereas the remaining 20 patients treated with tafamidis had either a percent decrease less than the median or even a percent increase from baseline and were assigned to *Cohort B* (*Figure 2B*).

### Baseline characteristics

Detailed baseline characteristics for the entire study population and for individual cohorts are depicted in *Table 1.* On average, ATTR-CM patients were 78.6 years (SD: 6.6) old and predominately male (82.5%). Median serum NT-proBNP levels were markedly elevated with 2181 pg/mL (IQR: 1248–3164) and comparable between the groups [2341 pg/mL (IQR: 1296–3164) vs. 1877 pg/mL (IQR: 920–3109), *P* = 0.800]. Nuclear imaging with ^99m^Tc-DPD scintigraphy of the total ATTR-CM cohort classified 47.5% patients with Perugini grade 2% and 52.5% with grade 3, without significant between-group differences (*Cohort A*, grade 2: 60.0%, grade 3: 40.0% vs. *Cohort B*, grade 2: 35.0%, grade 3: 65.0%; *P* = 0.119). Quantitative ^99m^Tc-DPD SPECT/CT imaging at baseline revealed a mean cardiac SUV peak of 14.64 g/mL (SD: 4.54) and a mean SUV retention index of 4.96 g/mL (SD: 2.46), which did not differ between patients in distinct cohorts (SUV peak cardiac: 14.82 g/mL ±3.80 vs. 14.45 g/mL ±5.28, *P* = 0.797; SUV retention index: 5.58 g/mL ±2.57 vs. 4.35 g/mL ±2.25, *P* = 0.117). Echocardiographic imaging of ATTR-CM patients showed LA enlargement [left atrial volume index (LAVI): 41.2 mL/m^2^ ± 15.1], which was comparable between groups (43.0 mL/m^2^ ± 16.2 vs. 39.6 mL/m^2^ ± 14.1, *P* = 0.488). Analysis of longitudinal function by 2D speckle-tracking echocardiography (STE) revealed impairments in LV global longitudinal strain (LV-GLS: −12.88% ± 3.14) and right ventricular (RV) longitudinal strain (RV-LS: −15.39% ± 5.37) that did not vary between cohorts (LV-GLS: −12.02% ± 3.21 vs. −13.91% ± 2.81, *P* = 0.095; RV-LS: −14.55% ± 4.81 vs. −16.38% ± 6.04, *P* = 0.418). ATTR-CM patients who underwent CMR (*n* = 25, Supplementary data online, *Table S1*) had mildly reduced LV function [LV ejection fraction (EF): 45.5% ± 10.2, LV cardiac index (CI): 2.68 L/min/m^2^ ± 0.65] and RV function (RVEF: 43.7% ± 10.2, RVCI: 2.35 L/min/m^2^ ± 0.51), which did not differ between cohorts (LVEF: 42.3% ± 10.4 vs. 48.5% ± 9.4, *P* = 0.133; LVCI: 2.63 L/min/m^2^ ± 0.69 vs. 2.75 L/min/m^2^ ± 0.38, *P* = 0.596; RVEF: 40.9% ± 9.2 vs. 46.2% ± 10.6, *P* = 0.193; RVCI: 2.29 L/min/m^2^ ± 0.64 vs. 2.41 L/min/m^2^ ± 0.36, *P* = 0.569) (*Figure 1*).

**Table 1:** Baseline characteristics

Characteristic	All patients (*n* = 40)	Cohort A (*n* = 20)	Cohort B (*n* = 20)	*P*-value
**Clinical parameters**				
Age (years), mean (SD)	78.6 (6.6)	80.5 (6.0)	76.6 (6.7)	0.060
Sex male, *n* (%)	33 (82.5)	17 (85.0)	16 (80.0)	0.687
Body mass index (kg/m^2^), mean (SD)	25.8 (3.7)	26.5 (3.4)	25.1 (3.9)	0.214
NYHA functional class ≥III, *n* (%)	22 (55.0)	14 (70.0)	8 (40.0)	0.059
6-min walk distance (m), mean (SD)	386.6 (130.8)	351.8 (132.5)	417.5 (124.9)	0.147
**Comorbidities, *n* (%)**				
Atrial fibrillation or flutter	20 (50.0)	10 (50.0)	10 (50.0)	1.000
Arterial hypertension	23 (57.5)	11 (55.0)	12 (60.0)	0.757
Coronary artery disease	13 (32.5)	8 (40.0)	5 (25.0)	0.324
Pacemaker or ICD	10 (25.0)	6 (30.0)	4 (20.0)	0.595
Polyneuropathy	24 (60.0)	12 (60.0)	12 (60.0)	1.000
Carpal tunnel syndrome	19 (47.5)	9 (45.0)	10 (50.0)	0.759
**Concomitant medication, *n* (%)**				
Anticoagulant	23 (57.5)	12 (60.0)	11 (55.0)	0.757
Beta-blocker	15 (37.5)	9 (45.0)	6 (30.0)	0.340
ACE inhibitor	13 (32.5)	7 (35.0)	6 (30.0)	0.744
Angiotensin receptor blocker	8 (20.0)	3 (15.0)	5 (25.0)	0.442
Diuretic agent	33 (82.5)	16 (80.0)	17 (85.0)	0.687
Mineralocorticoid receptor antagonist	21 (52.5)	9 (45.0)	12 (60.0)	0.355
**Laboratory parameters**				
Haemoglobin (g/dL), mean (SD)	13.5 (1.4)	13.1 (1.8)	13.8 (0.8)	0.106
Creatinine (mg/dL), mean (SD)	1.35 (0.80)	1.52 (1.07)	1.19 (0.30)	0.192
eGFR (mL/min/1.73 m^2^), mean (SD)	60.4 (21.6)	58.9 (26.0)	61.8 (16.7)	0.678
Troponin T (ng/L), mean (SD)	60.3 (53.6)	66.7 (72.4)	53.9 (23.7)	0.455
NT-proBNP (pg/mL), median (IQR)	2181 (1248–3164)	2341 (1296–3164)	1877 (920–3109)	0.800
**Nuclear imaging parameters**				
Perugini grade 2, *n* (%)	19 (47.5)	12 (60.0)	7 (35.0)	0.119
Perugini grade 3, *n* (%)	21 (52.5)	8 (40.0)	13 (65.0)	0.119
SUV peak cardiac (g/mL), mean (SD)	14.64 (4.54)	14.82 (3.80)	14.45 (5.28)	0.797
SUV retention index (g/mL), mean (SD)	4.96 (2.46)	5.58 (2.57)	4.35 (2.25)	0.117
^99m^Tc-DPD activity (MBq), mean (SD)	725.4 (25.7)	729.4 (24.8)	721.4 (26.6)	0.328
DLP (mGy*cm), mean (SD)	86.3 (30.7)	86.9 (26.9)	85.7 (34.9)	0.909
**Echocardiographic parameters**				
Intraventricular septum (mm), mean (SD)	19.2 (3.7)	19.9 (4.3)	18.5 (2.9)	0.234
LV end-diastolic diameter (mm), mean (SD)	43.0 (6.8)	43.9 (6.4)	42.2 (7.2)	0.430
LV ejection fraction (%), mean (SD)	49.1 (11.1)	47.3 (7.3)	50.6 (9.0)	0.231
LV global longitudinal strain (−%), mean (SD)	12.88 (3.14)	12.02 (3.21)	13.91 (2.81)	0.095
LA length (mm), mean (SD)	61.2 (9.2)	63.0 (11.1)	59.4 (6.6)	0.231
LA volume index (mL/m^2^), mean (SD)	41.2 (15.1)	43.0 (16.2)	39.6 (14.1)	0.488
LA reservoir strain (%), mean (SD)	9.15 (5.02)	8.18 (6.08)	9.97 (4.00)	0.398
RV end-diastolic diameter (mm), mean (SD)	33.5 (5.4)	35.8 (5.3)	31.4 (4.8)	**0**.**010**
RV longitudinal strain (−%), mean (SD)	15.39 (5.37)	14.55 (4.81)	16.38 (6.04)	0.418
RA length (mm), mean (SD)	59.6 (8.9)	59.7 (10.6)	59.6 (7.4)	0.949
TR velocity (m/s), mean (SD)	2.99 (0.44)	2.88 (0.41)	3.09 (0.46)	0.188
**CMR imaging parameters**	***n = 25***	***n = 12***	***n = 13***	
Interventricular septum (mm), mean (SD)	17.5 (3.5)	18.0 (3.1)	17.0 (4.0)	0.499
LV mass index (g/m^2^), mean (SD)	92.8 (23.2)	97.5 (23.2)	87.6 (23.0)	0.297
LV ejection fraction (%), mean (SD)	45.5 (10.2)	42.3 (10.4)	48.5 (9.4)	0.133
LV cardiac index (L/min/m^2^), mean (SD)	2.68 (0.65)	2.63 (0.69)	2.75 (0.38)	0.596
LA area index (cm^2^/m^2^), mean (SD)	15.7 (2.7)	16.4 (3.0)	15.1 (2.3)	0.239
RV ejection fraction (%), mean (SD)	43.7 (10.2)	40.9 (9.2)	46.2 (10.6)	0.193
RV cardiac index (L/min/m^2^), mean (SD)	2.35 (0.51)	2.29 (0.64)	2.41 (0.36)	0.569
RA area index (cm^2^/m^2^), mean (SD)	14.9 (4.1)	16.2 (4.7)	13.8 (3.2)	0.155
ECV (%), mean (SD)	50.2 (14.3)	53.4 (10.2)	47.0 (17.3)	0.286

Values are given as mean ± standard deviation (SD), or median and interquartile range (IQR), or total numbers (*n*) and percent (%). Bold indicates *P* < 0.05.

ACE, angiotensin-converting enzyme; CMR, cardiac magnetic resonance, DLP, dose length product; ECV, extracellular volume; eGFR, estimated glomerular filtration rate; ICD, implantable cardioverter defibrillator; LA, left atrium, LV, left ventricle; NT-proBNP, *N*-terminal prohormone of brain natriuretic peptide; NYHA, New York Heart Association; RA, right atrium; RV, right ventricle; SUV, standardized uptake value; TR, tricuspid regurgitation; ^99m^Tc-DPD, ^99m^Tc-3,3-diphosphono-1,2-propanodicarboxylic acid.

### Longitudinal changes in imaging parameters—within-cohort comparison

**Figure 3:**
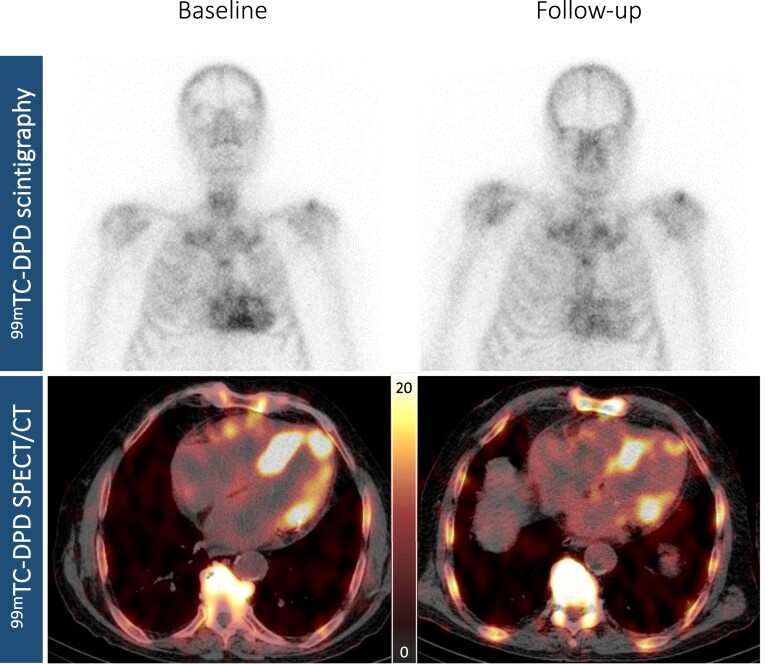
Visualization of response to tafamidis treatment by nuclear imaging. Planar whole-body ^99m^Tc-3,3-diphosphono-1,2-propanodicarboxylic acid (DPD) image (upper panels) and axial ^99m^Tc-DPD single-photon emission computed tomography/computed tomography (SPECT/CT) imaging (lower panels) of a representative tafamidis treatment-responsive patient with wild-type ATTR-CM at baseline (left panels, Perugini grade 3, SUV peak cardiac: 12.40 g/mL) and at follow-up (right panels, Perugini grade 2, SUV peak cardiac: 8.56 g/mL).

Detailed follow-up characteristics for the overall cohort and individual cohorts are shown in *Table 2*. ^99m^Tc-DPD scintigraphy and SPECT/CT follow-up were performed after a median of 9.0 months (IQR: 7.0–10.0). Using serial quantitative ^99m^Tc-DPD SPECT/CT imaging, we found a significant reduction in cardiac SUV peak (baseline: 14.64 g/mL vs. follow-up: 11.42 g/mL, *P* < 0.001, *Figure 3*) in the overall ATTR-CM cohort under tafamidis treatment, which is also evident when considering the individual cohorts (*Cohort A*: 14.82 g/mL vs. 10.77 g/mL, *P* < 0.001; *Cohort B*: 14.45 g/mL vs. 12.06 g/mL, *P* = 0.003). Adjustment with the SUV retention index revealed a significant decrease in the overall cohort (4.96 g/mL vs. 3.27 g/mL, *P* < 0.001) and *Cohort A* (5.58 g/mL vs. 2.66 g/mL, *P* < 0.001) at follow-up, while there was no improvement in *Cohort B* (4.35 g/mL vs. 3.87 g/mL, *P* = 0.116). Echocardiographically, we observed a significant decrease in LAVI (43.0 mL/m^2^ vs. 36.3 mL/m^2^, *P* = 0.046) in patients in *Cohort A*, but evidence of increase in LAVI (39.6 mL/m^2^ vs. 41.1 mL/m^2^, *P* = 0.507) in patients in *Cohort B*. Using 2D-STE, we found evidence of stabilization of LV-GLS (−12.02% vs. −12.44%, *P* = 0.520) and RV-LS (−14.55% vs. −13.85%, *P* = 0.580) in *Cohort A*, but significant impairments in LV (−13.91% vs. −12.01%, *P* = 0.030) and RV (−16.38% vs. −14.56%, *P* = 0.030) longitudinal functions in *Cohort B*. Analysis of cardiac function by CMR showed significant improvements in LV (LVEF: 42.3% vs. 48.0%, *P* = 0.047) and RV (RVEF: 40.9% vs. 46.2%, *P* = 0.036; RVCI: 2.29 L/min/m^2^ vs. 2.80 L/min/m^2^, *P* = 0.016) functions in ATTR-CM patients in *Cohort A*.

**Table 2:** Comparison of baseline and follow-up characteristics

Characteristic	All patients (*n* = 40)			Cohort A (*n* = 20)			Cohort B (*n* = 20)			Δ Cohort A	Δ Cohort B	
	Baseline	Follow-up	*P*-value	Baseline	Follow-up	*P*-value	Baseline	Follow-up	*P*-value			*P*-value
**Clinical parameters**												
Body mass index (kg/m^2^), mean (SD)	25.8 (3.7)	25.5 (3.5)	**0**.**011**	26.5 (3.4)	26.1 (3.4)	**0**.**029**	25.1 (3.9)	24.8 (3.6)	0.166	−0.4 (0.7)	−0.3 (0.8)	0.626
NYHA functional class ≥III, *n* (%)	22 (55.0)	17 (42.5)	**0**.**030**	14 (70.0)	9 (45.0)	**0**.**021**	8 (40.0)	8 (40.0)	1.000	−5 (25.0)	0 (0.0)	**0**.**049**
6-min walk distance (m), mean (SD)	386.6 (130.8)	380.2 (145.3)	0.631	351.8 (132.5)	364.6 (158.6)	0.556	417.5 (124.9)	394.1 (135.4)	0.148	12.8 (85.2)	−23.4 (65.5)	0.172
**Laboratory parameters**												
Haemoglobin (g/dL), mean (SD)	13.5 (1.4)	13.4 (1.4)	0.414	13.1 (1.8)	13.1 (1.8)	1.000	13.8 (0.8)	13.6 (0.8)	0.224	0.0 (0.8)	−0.2 (0.7)	0.416
Creatinine (mg/dL), mean (SD)	1.35 (0.80)	1.43 (0.93)	0.298	1.52 (1.07)	1.63 (1.27)	0.442	1.19 (0.30)	1.23 (0.28)	0.311	0.11 (0.63)	0.04 (0.18)	0.643
eGFR (mL/min/1.73m^2^), mean (SD)	60.4 (21.6)	57.0 (19.8)	0.052	58.9 (26.0)	54.9 (24.3)	0.181	61.8 (16.7)	59.0 (14.4)	0.136	−4.0 (12.8)	−2.8 (8.0)	0.730
Troponin T (ng/L), mean (SD)	60.3 (53.6)	63.9 (53.8)	0.094	66.7 (72.4)	69.7 (71.1)	0.251	53.9 (23.7)	58.2 (28.7)	0.231	3.0 (11.1)	4.3 (15.5)	0.754
NT-proBNP (pg/mL), median (IQR)	2181 (1248–3164)	1675 (1212–2682)	0.068	2341 (1296–3164)	1492 (1263–2148)	**0**.**002**	1877 (920–3109)	1943 (1202–3010)	0.550	−849	66	**0**.**006**
**Nuclear imaging parameters**												
Perugini grade 3, *n* (%)	21 (52.5)	18 (45.0)	0.262	8 (40.0)	5 (25.0)	0.186	13 (65.0)	13 (65.0)	1.000	−3 (15.0)	0 (0.0)	0.657
SUV peak cardiac (g/mL), mean (SD)	14.64 (4.54)	11.42 (3.94)	**<0**.**001**	14.82 (3.80)	10.77 (3.98)	**<0**.**001**	14.45 (5.28)	12.06 (3.90)	**0**.**003**	−4.05 (2.43)	−2.39 (3.09)	0.066
SUV retention index (g/mL), mean (SD)	4.96 (2.46)	3.27 (1.77)	**<0**.**001**	5.58 (2.57)	2.66 (1.42)	**<0**.**001**	4.35 (2.25)	3.87 (1.92)	0.116	−2.92 (1.80)	−0.48 (1.31)	**<0**.**001**
^99m^Tc-DPD activity (MBq), mean (SD)	725.4 (25.7)	720.8 (28.7)	0.452	729.4 (24.8)	723.9 (38.6)	0.615	721.4 (26.6)	717.7 (13.6)	0.531	−5.5 (49.0)	−3.7 (25.9)	0.879
DLP (mGy*cm), mean (SD)	86.3 (30.7)	91.9 (40.1)	0.407	86.9 (26.9)	98.2 (37.5)	0.190	85.7 (34.9)	85.2 (42.7)	0.959	11.3 (37.4)	−0.5 (45.7)	0.377
**Echocardiographic parameters**												
Intraventricular septum (mm), mean (SD)	19.2 (3.7)	19.9 (3.8)	**0**.**010**	19.9 (4.3)	20.5 (4.4)	0.077	18.5 (2.9)	19.4 (2.9)	0.061	0.6 (1.3)	0.9 (2.1)	0.476
LV end-diastolic diameter (mm), mean (SD)	43.0 (6.8)	41.4 (8.0)	0.116	43.9 (6.4)	42.2 (8.1)	0.246	42.2 (7.2)	40.7 (8.1)	0.307	−1.7 (6.3)	−1.5 (10.0)	0.908
LV ejection fraction (%), mean (SD)	49.1 (11.1)	48.9 (8.2)	0.923	47.3 (7.3)	49.8 (12.6)	0.306	50.6 (9.0)	48.3 (9.6)	0.153	2.5 (10.0)	−2.3 (6.5)	0.101
LV global longitudinal strain (−%), mean (SD)	12.88 (3.14)	12.25 (3.94)	0.251	12.02 (3.21)	12.44 (3.91)	0.520	13.91 (2.81)	12.01 (4.12)	**0**.**030**	0.42 (2.66)	−1.90 (2.92)	**0**.**028**
LA length (mm), mean (SD)	61.2 (9.2)	60.5 (7.4)	0.651	63.0 (11.1)	59.1 (7.3)	0.072	59.4 (6.6)	61.9 (7.3)	0.134	−3.9 (8.9)	2.5 (7.1)	**0**.**018**
LA volume index (mL/m^2^), mean (SD)	41.2 (15.1)	38.8 (11.8)	0.220	43.0 (16.2)	36.3 (11.3)	**0**.**046**	39.6 (14.1)	41.1 (12.1)	0.507	−6.7 (13.6)	1.5 (10.0)	**0**.**038**
LA reservoir strain (%), mean (SD)	9.15 (5.02)	8.66 (4.55)	0.438	8.18 (6.08)	8.47 (5.43)	0.461	9.97 (4.00)	8.82 (3.88)	0.313	0.29 (1.23)	−1.15 (3.92)	0.233
RV end-diastolic diameter (mm), mean (SD)	33.5 (5.4)	34.5 (4.3)	0.198	35.8 (5.3)	35.7 (4.2)	0.959	31.4 (4.8)	33.4 (4.2)	0.089	−0.1 (4.4)	2.0 (5.0)	0.182
RV longitudinal strain (−%), mean (SD)	15.39 (5.37)	14.18 (5.27)	0.113	14.55 (4.81)	13.85 (4.99)	0.580	16.38 (6.04)	14.56 (5.80)	**0**.**030**	−0.70 (4.42)	−1.82 (2.38)	0.459
RA length (mm), mean (SD)	59.6 (8.9)	59.7 (7.3)	0.939	59.7 (10.6)	58.5 (7.2)	0.506	59.6 (7.4)	60.9 (7.4)	0.091	−1.2 (8.1)	1.3 (3.4)	0.206
TR velocity (m/s), mean (SD)	2.99 (0.44)	3.01 (0.39)	0.684	2.88 (0.41)	2.89 (0.36)	0.902	3.09 (0.46)	3.12 (0.40)	0.661	0.01 (0.34)	0.03 (0.33)	0.830
**CMR imaging parameters**	***n = 25***			***n = 12***			***n = 13***					
Intraventricular septum (mm), mean (SD)	17.5 (3.5)	18.1 (3.2)	0.162	18.0 (3.1)	18.1 (2.9)	0.767	17.0 (4.0)	18.0 (3.6)	0.103	0.1 (1.8)	1.0 (1.8)	0.296
LV mass index (g/m^2^), mean (SD)	92.8 (23.2)	94.5 (23.5)	0.620	97.5 (23.2)	96.4 (2.0.5)	0.858	87.6 (23.0)	92.5 (27.1)	0.169	−1.1 (21.7)	4.9 (11.4)	0.405
LV ejection fraction (%), mean (SD)	45.5 (10.2)	47.0 (8.2)	0.443	42.3 (10.4)	48.0 (8.0)	**0**.**047**	48.5 (9.4)	46.0 (8.6)	0.295	5.7 (9.2)	−2.5 (8.1)	**0**.**027**
LV cardiac index (L/min/m^2^), mean (SD)	2.68 (0.65)	2.69 (0.55)	0.906	2.63 (0.69)	2.89 (0.62)	0.154	2.75 (0.38)	2.47 (0.64)	0.124	0.26 (0.60)	−0.28 (0.60)	**0**.**034**
LA area index (cm^2^/m^2^), mean (SD)	15.7 (2.7)	16.1 (3.0)	0.369	16.4 (3.0)	16.8 (2.4)	0.168	15.1 (2.3)	15.4 (3.4)	0.686	0.4 (1.1)	0.3 (2.9)	0.876
RV ejection fraction (%), mean (SD)	43.7 (10.2)	44.2 (11.1)	0.812	40.9 (9.2)	46.2 (12.0)	**0**.**036**	46.2 (10.6)	42.3 (10.3)	0.227	5.3 (7.7)	−3.9 (11.1)	**0**.**025**
RV cardiac index (L/min/m^2^), mean (SD)	2.35 (0.51)	2.60 (0.57)	0.069	2.29 (0.64)	2.80 (0.56)	**0**.**016**	2.41 (0.36)	2.42 (0.53)	0.978	0.51 (0.63)	0.01 (0.60)	**0**.**048**
RA area index (cm^2^/m^2^), mean (SD)	14.9 (4.1)	15.4 (4.4)	0.315	16.2 (4.7)	16.8 (4.9)	0.285	13.8 (3.2)	14.2 (3.6)	0.611	0.6 (1.9)	0.4 (3.2)	0.892
ECV (%), mean (SD)	50.2 (14.3)	52.8 (16.6)	0.169	53.4 (10.2)	54.5 (10.7)	0.720	47.0 (17.3)	51.2 (21.4)	0.103	1.1 (10.0)	4.2 (8.2)	0.408

Values are given as mean ± standard deviation (SD), or median and interquartile range (IQR), or total numbers (*n*) and percent (%). Bold indicates *P* < 0.05. Abbreviations as in *Table 1*.

### Longitudinal changes in imaging and clinical parameters—between-cohort comparison

**Figure 4:**
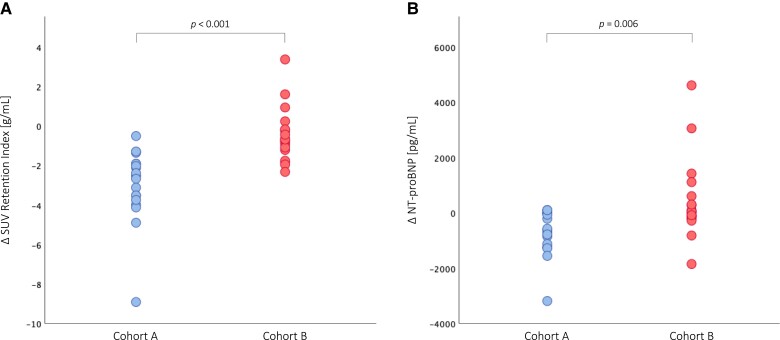
Longitudinal changes in nuclear imaging and clinical parameters. (*A*) Change in standardized uptake value (SUV) retention index. (*B*) Change in serum *N*-terminal prohormone of brain natriuretic peptide (NT-proBNP) levels.

**Figure 5:**
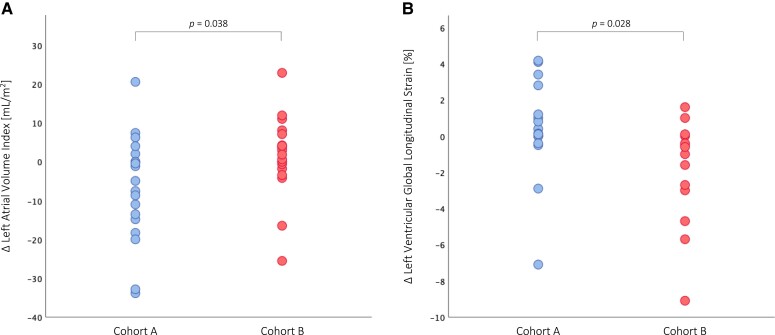
Longitudinal changes in echocardiographic parameters. (*A*) Change in left atrial volume index. (*B*) Change in left ventricular global longitudinal strain.

**Figure 6:**
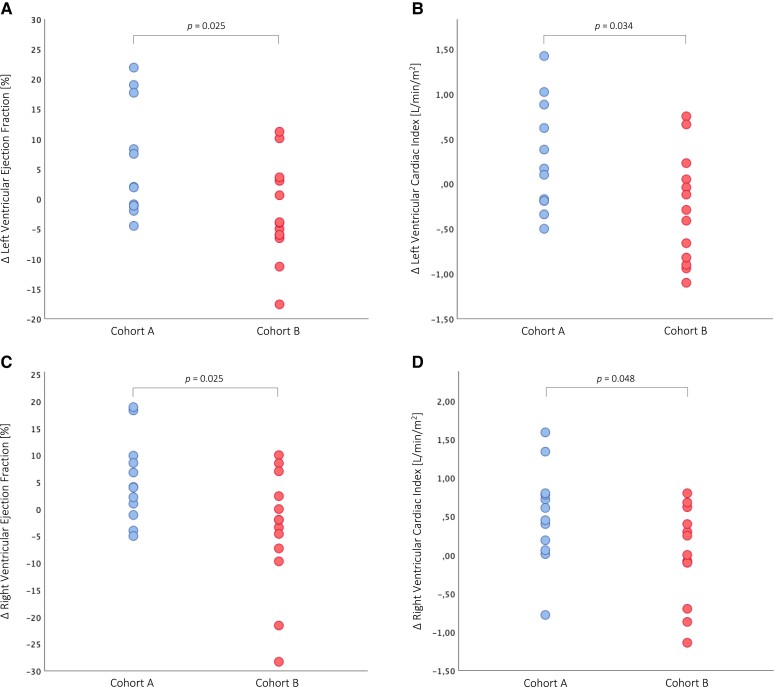
Longitudinal changes in cardiac magnetic resonance imaging parameters. (*A*) Change in left ventricular ejection fraction. (*B*) Change in left ventricular cardiac index. (*C*) Change in right ventricular ejection fraction. (*D*) Change in right ventricular cardiac index.

ATTR-CM patients in *Cohort A* showed a statistically significant decrease in SUV retention index (*P* < 0.001, *Figure 4A*) at the end of the observation period, which translated into significant benefits in serum NT-proBNP levels (*P* = 0.006, *Figure 4B*), LAVI (*P* = 0.038, *Figure 5A*), as well as LV function (LV-GLS: *P* = 0.028, *Figure 5B*; LVEF: *P* = 0.027, *Figure 6A*; LVCI: *P* = 0.034, *Figure 6B*) and RV function (RVEF: *P* = 0.025, *Figure 6C*; RVCI: *P* = 0.048, *Figure 6D*) compared with patients in *Cohort B*.

## Discussion

Treatment with tafamidis positively affects LV structure and function and improves outcomes in ATTR-CM patients.^7,8^ Novel nuclear imaging techniques such as quantitative SPECT/CT imaging may contribute to a better understanding of the relationship between tafamidis treatment and response and may therefore be suitable for monitoring disease-specific therapies.^6^ Using serial quantitative ^99m^TC-DPD SPECT/CT imaging with SUV, we were able to demonstrate that (i) treatment with tafamidis 61 mg QD in ATTR-CM patients resulted in a significant decrease in cardiac SUV peak and SUV retention index from baseline; (ii) ATTR-CM patients in whom the percent decrease in SUV retention index was greater than or equal to the median had significant benefits in serum NT-proBNP levels, LAVI, as well as LV and RV function; (iii) serial quantitative ^99m^TC-DPD-SPECT/CT imaging with SUV may be a valid tool for quantifying and monitoring disease-specific therapy in affected patients.

In the present study, ATTR-CM patients (*n* = 40) treated with tafamidis 61 mg QD for a median of 9.0 (IQR 7.0–10.0) months showed a significant decrease in cardiac SUV peak (*P* < 0.001) and SUV retention index (*P* < 0.001) compared with baseline, which is consistent with previous findings.^21,22^ Moreover, ATTR-CM patients in whom the percent decrease in SUV retention index was greater than or equal to the median (*Cohort A*, *n* = 20) had a significant reduction in SUV retention index (*P* < 0.001) at the end of the observation period, while patients in whom the percent decrease was less than the median (*Cohort B*, *n* = 20) showed no significant improvement at follow-up (*P* = 0.116), resulting in significant differences in cohort comparison (*P* < 0.001, *Figure 4A*).

When comparing the two cohorts on a clinical level, we observed a significant improvement in serum NT-proBNP levels in patients in *Cohort A* (*P* = 0.002) as opposed to *Cohort B* (*P* = 0.550; cohort comparison: *P* = 0.006, *Figure 4B*). These findings suggest that the reduction in SUV retention index induced by tafamidis is associated with clinical benefits and may be reflected in clinical outcomes.

Further evidence that the tafamidis-induced decrease in SUV retention index is associated with beneficial effects was provided by imaging of the LV. ATTR-CM patients in *Cohort A* had a significant improvement in LVEF (*P* = 0.047) and evidence of LV-GLS stabilization (*P* = 0.520), while patients in *Cohort B* showed no beneficial effect on LVEF (*P* = 0.295, cohort comparison: *P* = 0.027, *Figure 6A*) and experienced a significant worsening of LV longitudinal function at follow-up (*P* = 0.030, cohort comparison: *P* = 0.028, *Figure 5B*). These results are consistent with recently published echocardiographic data describing less deterioration of LV-GLS with tafamidis treatment.^23,24^ In addition, we observed significant benefits in LVCI in patients of *Cohort A* compared with patients in *Cohort B* (*P* = 0.034, *Figure 6B*), which is further supported by the results of our CMR imaging study, in which a beneficial effect of tafamidis treatment on LV function was observed.^7^

When focusing on the RV, we found evidence of stabilization of RV-LS in ATTR-CM patients in *Cohort A* (*P* = 0.580), while patients in *Cohort B* experienced a significant deterioration of RV longitudinal function (*P* = 0.030). This is also evident in the assessment of RV function by CMR, which showed significant benefits in patients in *Cohort A* compared with *Cohort B* (RVEF: *P* = 0.025, *Figure 6C*; RVCI: *P* = 0.048, *Figure 6D*). Interestingly, beneficial effects of tafamidis on RV function have not yet been reported in the literature. However, in ATTR-CM, amyloid fibrils can also be expected to be deposited in the RV,^25^ as shown by the tracer accumulation in *Figure 3*, demonstrating improvements in the LV and RV in *Cohort A* at follow-up. Therefore, tafamidis-induced reduction in cardiac SUV peak and SUV retention index in *Cohort A* may affect both LV and RV, as reflected by significant improvements in LVEF (*P* = 0.057) and RVEF (*P* = 0.036) in the CMR cohort.

Nevertheless, there is still uncertainty about the mechanisms underlying the beneficial response to tafamidis treatment and whether there are differences in outcomes between patients in *Cohort A* and *Cohort B*. Further long-term studies to evaluate the association between tafamidis-induced reduction in SUV retention index and outcome are warranted and will demonstrate whether highly disease-specific ^99m^Tc-DPD SPECT/CT imaging is more sensitive than routine diagnostic monitoring.

### Limitations

Several limitations are inherent to the present study. First, the present study is limited by its sample size due to the single-centre design, and centre-specific bias cannot be excluded. However, limiting data collection to one centre has the advantages of constant quality of work-up, adherence to a constant clinical routine, and constant follow-up. Second, individual differences in the duration of tafamidis treatment depending on the timing of ^99m^TC-DPD SPECT/CT follow-up, as well as differences between timing of baseline TTE, CMR, and ^99m^TC-DPD SPECT/CT imaging and follow-up may have affected the results. However, this is the first study to systematically perform serial quantitative ^99m^TC-DPD SPECT/CT imaging with clinical and imaging outcomes in ATTR-CM patients treated with tafamidis. Third, the significant beneficial effects on LVEF observed with CMR in *Cohort A* were not reflected in TTE, indicating a small observed effect. However, CMR is the established gold standard for quantification of EF.^26^ Fourth, although there is evidence that cardiac SUV peak correlates with ECV^14^ and thus with amyloid burden in the myocardium,^27^ this has not yet been histologically validated. However, the study’s main findings are consistent with previously published data. Finally, the observation period was too short to assess differences in outcome between cohorts.

## Conclusion

Using serial quantitative ^99m^Tc-DPD SPECT/CT imaging with SUV, we demonstrated that treatment with tafamidis 61 mg QD in ATTR-CM patients results in a significant reduction in SUV retention index, associated with significant benefits for LV and RV function and cardiac biomarkers. Our data suggest that serial quantitative ^99m^Tc-DPD SPECT/CT imaging with SUV may be a valid tool to quantify and monitor response to tafamidis treatment in affected patients.

## Supplementary data

Supplementary data are available at *European Heart Journal - Cardiovascular Imaging* online.

## Supplementary Material

jead030_Supplementary_Data

## Data Availability

The data underlying this article are available in the article. There is no online supplementary material.
