# Supplementary material for: Monitoring tafamidis treatment with quantitative SPECT/CT in transthyretin amyloid cardiomyopathy
Source: Eur Heart J Cardiovasc Imaging. 2023 Mar 7;24(8):1019–30. doi: 10.1093/ehjci/jead030 (PMC10364619; doi:10.1093/ehjci/jead030)
Supplement: jead030_Supplementary_Data [file jead030_supplementary_data.docx]

**Supplementary Table 1.** Baseline Characteristics – Comparison of Cardiac Magnetic Resonance Imaging and Non Cardiac Magnetic Resonance Imaging Cohorts.

| *Characteristic* | *All Patients (n=40)* | *CMR Cohort (n=25)* | *Non-CMR Cohort (n=15)* | *p-Value* |
| --- | --- | --- | --- | --- |
| Clinical Parameters |  |  |  |  |
| Age (years), mean (SD) | 78.6 (6.6) | 78.9 (5.2) | 77.0 (8.6) | 0.652 |
| Sex male, n (%) | 33 (82.5) | 20 (80.0) | 13 (86.7) | 0.602 |
| Body-mass index (kg/m^2^), mean (SD) | 25.8 (3.7) | 26.0 (3.4) | 25.5 (4.3) | 0.717 |
| NYHA functional class ≥ III, n (%) | 22 (55.0) | 13 (52.0) | 9 (60.0) | 0.463 |
| 6-min walk distance (m), mean (SD) | 386.6 (130.8) | 396.8 (139.1) | 370.1 (119.7) | 0.571 |
| Comorbidities, n (%) |  |  |  |  |
| Atrial fibrillation or flutter | 20 (50.0) | 9 (36.0) | 11 (73.3) | **0.022** |
| Arterial hypertension | 17 (42.5) | 17 (68.0) | 6 (40.0) | 0.087 |
| Coronary artery disease | 13 (32.5) | 9 (36.0) | 4 (26.7) | 0.554 |
| Pacemaker or ICD | 10 (25.0) | 0 (0.0) | 10 (66.7) | **<0.001** |
| Polyneuropathy | 24 (60.0) | 15 (60.0) | 9 (60.0) | 1.000 |
| Carpal tunnel syndrome | 19 (47.5) | 14 (56.0) | 5 (33.3) | 0.173 |
| Concomitant Medication, n (%) |  |  |  |  |
| Anticoagulant | 23 (57.5) | 10 (40.0) | 13 (86.7) | **0.001** |
| Beta-blocker | 15 (37.5) | 8 (32.0) | 7 (46.7) | 0.366 |
| ACE inhibitor | 13 (32.5) | 9 (36.0) | 4 (26.7) | 0.554 |
| Angiotensin receptor blocker | 8 (20.0) | 7 (28.0) | 1 (6.7) | 0.067 |
| Diuretic agent | 33 (82.5) | 20 (80.0) | 13 (86.7) | 0.602 |
| Mineralocorticoid receptor antagonist | 21 (52.5) | 12 (48.0) | 9 (60.0) | 0.475 |
| Laboratory Parameters |  |  |  |  |
| Hemoglobin (g/dL), mean (SD) | 13.5 (1.4) | 13.7 (1.1) | 13.1 (1.9) | 0.298 |
| Creatinine (mg/dL), mean (SD) | 1.35 (0.80) | 1.22 (0.34) | 1.58 (1.22) | 0.284 |
| eGFR (mL/min/1.73m^2^), mean (SD) | 60.4 (21.6) | 61.1 (16.9) | 59.1 (28.4) | 0.813 |
| Troponin T (ng/L), mean (SD) | 60.3 (53.6) | 48.0 (21.0) | 80.8 (80.7) | 0.143 |
| NT-proBNP (pg/mL), median (IQR) | 2181  (1248-3164) | 1876  (933-2928) | 2495 (1510-3164) | 0.320 |
| Nuclear Imaging Parameters |  |  |  |  |
| Perugini grade 2, n (%) | 19 (47.5) | 11 (44.0) | 8 (53.3) | 0.579 |
| Perugini grade 3, n (%) | 21 (52.5) | 14 (56.0) | 7 (46.7) | 0.579 |
| SUV peak cardiac (g/mL), mean (SD) | 14.64 (4.54) | 14.68 (4.88) | 14.57 (4.08) | 0.941 |
| SUV retention index (g/mL), mean (SD) | 4.96 (2.46) | 4.87 (2.24) | 5.12 (2.87) | 0.760 |
| ^99m^Tc-DPD activity (MBq), mean (SD) | 725.4 (25.7) | 722.7 (27.4) | 729.9 (22.8) | 0.397 |
| DLP (mGy*cm), mean (SD) | 86.3 (30.7) | 89.2 (32.2) | 81.6 (28.5) | 0.453 |
| Echocardiographic Parameters |  |  |  |  |
| Intraventricular septum (mm), mean (SD) | 19.2 (3.7) | 18.8 (3.1) | 20.0 (4.6) | 0.322 |
| LV end-diastolic diameter (mm), mean (SD) | 43.0 (6.8) | 42.4 (7.0) | 44.1 (6.6) | 0.468 |
| LV ejection fraction (%), mean (SD) | 49.1 (11.1) | 49.2 (8.5) | 48.4 (8.1) | 0.775 |
| LV global longitudinal strain (-%), mean (SD) | 12.88 (3.14) | 13.58 (3.29) | 11.59 (2.48) | 0.069 |
| LA length (mm), mean (SD) | 61.2 (9.2) | 60.9 (8.6) | 61.7 (10.4) | 0.815 |
| LA volume index (mL/m^2^), mean (SD) | 41.2 (15.1) | 40.8 (16.9) | 42.1 (11.8) | 0.804 |
| LA reservoir strain (%), mean (SD) | 9.15 (5.02) | 9.75 (5.64) | 7.33 (1.61) | 0.117 |
| RV end-diastolic diameter (mm), mean (SD) | 33.5 (5.4) | 32.5 (5.9) | 35.4 (3.9) | 0.104 |
| RV longitudinal strain (-%), mean (SD) | 15.39 (5.37) | 13.57 (4.58) | 17.43 (6.73) | 0.240 |
| RA length (mm), mean (SD) | 59.6 (8.9) | 59.2 (8.1) | 60.5 (10.5) | 0.660 |
| TR velocity (m/s), mean (SD) | 2.99 (0.44) | 2.9 (0.5) | 3.1 (0.4) | 0.187 |

Values are given as mean ± standard deviation (SD), or median and interquartile range (IQR), or total numbers (n) and percent (%). Bold indicates *p* <0.05.

ACE = Angiotensin-converting enzyme, CMR = Cardiac magnetic resonance, DLP = Dose length product, eGFR = estimated glomerular filtration rate, ICD = Implantable Cardioverter Defibrillator, LA = Left atrium, LV = Left ventricle, NT-proBNP = N-terminal prohormone of brain natriuretic peptide, NYHA = New York Heart Association, RA = Right atrium, RV = Right ventricle, SUV = Standardized uptake value, TR = Tricuspid regurgitation, ^99m^Tc-DPD = ^99m^Tc-3,3-diphosphono-1,2-propanodicarboxylic acid.
